# Retraction of: Genome Report: Long read, high-coverage reference genomes of the Nymphalid butterflies *Catonephele acontius* and *Catonephele numilia* (Nymphalidae: Biblidinae)

**DOI:** 10.1093/g3journal/jkag051

**Published:** 2026-03-19

This is a retraction of: Marcus Hicks, Tan Nhat Pham, Océane Seudre, Zunilda Escalante, Lucy S Knowles, Geoffrey Gallice, Vicencio Oostra, Genome Report: Long read, high-coverage reference genomes of the Nymphalid butterflies *Catonephele acontius* and *Catonephele numilia* (Nymphalidae: Biblidinae), *G3 Genes|Genomes|Genetics*, 2026, jkag029, https://doi.org/10.1093/g3journal/jkag029

In February 2026, the authors notified the Editorial Office that the *Catonephele numilia* specimen was misidentified and was in fact also *Catonephele acontius*. Thus, the data in the article describing the *Catonephele numilia* genome is invalid, as it in fact describes a second *Catonephele acontius* genome. This data invalidation has resulted in a loss of confidence in the results and conclusions reported in the article as far as they apply to *Catonephele numilia*. The data describing *Catonephele acontius* is not affected and remains valid. The authors and Editors are, therefore, retracting this article. All authors agree with the retraction.

